# Presidential address: 61^st^ National Conference of the Indian Radiological and Imaging Association (IRIA), Bangalore

**Published:** 2008-05

**Authors:** N Kulasekaran


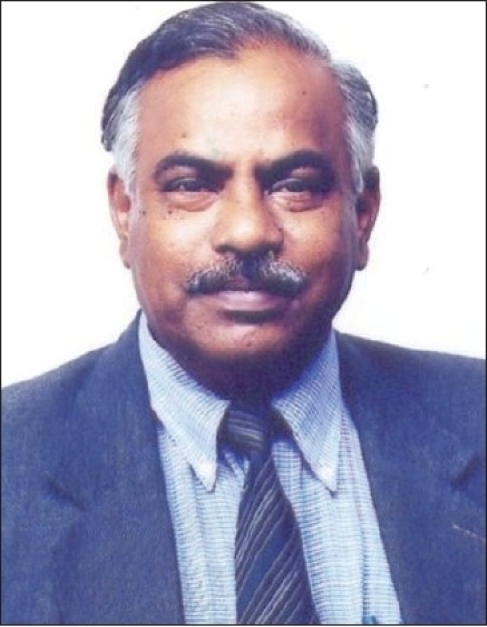


Esteemed Chief Guest, His Excellency, The Governor of Karnataka, Mr. Rameshwar Thakur, the outgoing president and my close friend, Prof. Satishchandra, respected dignitaries on the dais and off the dais, senior professors, distinguished colleagues, friends and dearest residents.

It is a great privilege and honor to be standing here as the newly inducted President of the Indian Radiological and Imaging association (IRIA) for the year 2008, addressing this elite gathering.

I thank each and every one of you for having had so much confidence in me, and I assure you that I will strive to fulfill all your wishes and aims so as to make our field as outstanding as possible.

Like me, I am sure that each one of you is proud to be a radiologist. Moreover, radiology is the most demanding branch in the medical field right now. It is advancing by leaps and bounds, be it in technology, equipment or applications. But at this point, we also have to admit a sorry fact; we do have a lacuna as far as expense is concerned. Everything in radiology is exorbitantly expensive and it is only with the help of our Government that the cost of equipments, which are life-saving, can be reduced by slashing down import duties and other taxes and also increasing the manpower proportionately so as to make the benefits of imaging universally available.

Talking about the manpower, there is undoubtedly an acute scarcity of radiologists in our country. For our immense and ever-growing population, a minimum of about one lakh radiologists are needed for adequate service. You will be surprised to know, that at present we have only 6000 plus registered radiologists in our society and not more than 10,000 in and around our country. Consequently, our work is not as evenly spread out as it should be, as in the rest of the developed countries. We need the Medical Council, National Board of Examinations and Government of India to put in their best and untiring efforts to increase the number of postgraduate seats in radiodiagnosis, thereby increasing the strength of radiologists, so as to make this modality reach into all the nook and corners of our country.

Another vital request that I need to put in at this juncture, the branch of radiodiagnosis remains the only discipline recognized by Universities, authorized to practice ultrasound. With spurts of sonologists and even sonographers cropping up everywhere, the practice of ultrasound is becoming nothing but a *qualified quackery system*, which can be tackled by the improved strength of radiologists.

It is a paradox, that when some of us have to go through 2-3 years of grilling to get ourselves qualified to practice CT, MR or ultrasound, there are certain people who undergo a month or even just 2 weeks of so-called “ultrasound training” and start doing full-fledged sonography. “Isn't this a pathetic situation?” At this juncture, I sincerely request our colleagues to refrain from training non-radiologists in the field of ultrasound so that we can limit this *qualified quackery system*.

We have to unite and try to make ultrasound practice as ethical and organized as possible. With this in mind, I will take a moment to discuss the PNDT act. Every unit that practices ultrasound needs to have prior PNDT approval. This makes the governing authority decide as to which center can go ahead and practice ultrasound and which cannot. It is, therefore, essential that the radiologists should be on the board of committees and subcommittees of the PNDT act. After all, we are the only ones with an in-depth knowledge and expertise in the practice of ultrasound. We can guide the committees as to how to draft and put into action the rules and regulations regarding ultrasound practice. It would also be preferable to hold an orientation course for the members of these committees. This would help them to get a clear idea as to their duties and the various lacunae, which need to be filled. I strongly believe that this would reduce the commercialized ultrasound practice by non-radiologists.

One more thought, utmost in my agenda now, is the way our radiology society has branched out into various societies individually as per subspecialties. We have annual conventions being held separately for vascular and interventional radiology, neuroradiology, pediatric radiology, nuclear medicine, so on and so forth. It is a long-term dream of mine to integrate all these societies under one parent organization - the one and only Indian Radiological and Imaging Association like Radiological Society of North America (RSNA), European Society of Radiology (ESR), AOSR, CSR, etc. If all these radiology suborganizations can be gathered under the wings of IRIA, while maintaining their individual identities, like Indian College of Radiology and Imaging (ICRI), it would make a strong and united front for all of us. The conventions of all societies of the radiological and imaging sciences can be brought under one roof during the National Annual Congress of the IRIA, which will benefit all our members and also the healthcare industries. For this is a well-known fact: “United we stand, Divided we fall”.

Further, we should adequately train and refresh the knowledge of our residents and practicing radiologists by conducting several CME programs in the nook and corner of our country through our state chapters and city/town subchapters. For this, we should encourage the state chapters to create more city/town subchapters in their states. In addition, our academic wing, the ICRI, should take responsibility to organize the CME programs evenly throughout the country to train and upgrade the knowledge of our residents and the practicing radiologists.

This is only the beginning. I have a long way to go in a short period of 1 year and a lot more to accomplish. The facts I have outlined to you so far are only the tip of the iceberg. There are so many smaller things I need to do and I am sure, with your esteemed co-operation, this is going to be yet another successful and fruitful year for the IRIA. At this juncture, I thank all the past presidents who have laid this successful foundation and have guided us in this direction for the development of our society to the highest peak.

Long Live IRIA.

Thank you very much.

Dr. N. Kulasekaran (17^th^ January 2008)

